# Experimental–Numerical Framework for Evaluating the Mechanical Response of *Cornus sanguinea* L.-Reinforced Polypropylene Biocomposites

**DOI:** 10.3390/polym18091020

**Published:** 2026-04-23

**Authors:** Mustafa Öncül

**Affiliations:** Department of Mechanical Engineering, İzmir Katip Çelebi University, 35620 İzmir, Türkiye; mustafa.oncul@ikcu.edu.tr

**Keywords:** polypropylene biocomposites, sustainable lignocellulosic fillers, agricultural waste valorization, finite element modeling, structure-property relationships

## Abstract

Polypropylene (PP) biocomposites reinforced with *Cornus sanguinea* L. (CS) pruning-waste particles were investigated using a combined experimental mechanics and finite element (FE) validation framework to support model-based design with an under-utilized lignocellulosic feedstock. Two particle-size fractions (<100 µm, LF1; 100–250 µm, LF2) were produced by grinding and sieving and incorporated into PP at 5–20 wt% via melt compounding and compression molding. Tensile and three-point bending properties were measured in accordance with ASTM D638 and ASTM D790. PP exhibited a tensile strength of 23.63 ± 0.51 MPa and a tensile modulus of 868 ± 21 MPa. Incorporation of LF1 particles increased tensile modulus monotonically, reaching 1020 ± 137 MPa at 20 wt%, while tensile strength decreased with filler content; by contrast, the 20 wt% LF2 formulation showed a pronounced strength reduction to 16.30 ± 0.25 MPa, indicating a disadvantageous size–loading interaction. In flexure, strength was comparatively insensitive to reinforcement (PP: 39.5 ± 0.34 MPa; reductions typically ≤7%), whereas flexural modulus increased to 2152 ± 27 MPa (LF1) and 2110 ± 34 MPa (LF2). FE models calibrated using true stress–true plastic strain data accurately reproduced tensile responses across the full strain range and flexural behavior within the pre-contact-dominated regime, demonstrating the suitability of PP/CS biocomposites for stiffness-driven applications.

## 1. Introduction

Wood–plastic composites (WPCs) and related natural-fiber-reinforced thermoplastics are increasingly developed to reduce cost, density, and environmental impact while retaining stiffness and serviceable durability in semi-structural applications [1,2,3,4,5]. Among commodity matrices, polypropylene (PP) remains particularly attractive due to its balanced processability, recyclability, and mechanical performance. Prior studies consistently show that lignocellulosic fillers increase elastic modulus in PP, whereas strength and ductility are governed by particle size, loading level, dispersion quality, and interfacial compatibility [2,6,7,8]. From an engineering perspective, these trends highlight a persistent stiffness–strength trade-off that must be addressed if biocomposites are to be integrated into mechanically loaded components rather than limited to non-structural uses.

Beyond conventional wood sources, pruning waste from under-utilized plant species has emerged as a promising feedstock for sustainable composite systems, provided that its mechanical implications are rigorously quantified and transferable design data can be established [9,10,11,12]. For instance, it was demonstrated that the incorporation of pruned cherry tree branch wood and bark particles into PP increased storage and tensile moduli but reduced tensile strength at higher filler contents in the absence of coupling agents [6]. This behavior highlights the sensitivity of stress transfer to particle size and interfacial conditions. Comparable behavior has been reported in thermoset systems [13,14,15], as well as in studies on less explored fibers, such as Encephalartos ituriensis, and untreated PLA-based biocomposites [16,17]. These studies further reinforce the generality of this response across polymer matrices: modulus increases monotonically with filler content, while tensile strength and elongation decrease beyond approximately 10 wt%.

Collectively, these results indicate that alternative biomass sources are mechanically viable, yet systematic investigations linking particle-size distributions to engineering response remain limited. To address this gap and advance sustainable manufacturing, *Cornus sanguinea* L. (CS) was selected as the natural filler in this research. This selection was primarily motivated by the species’ widespread regional abundance and the significant volume of agricultural pruning waste it generates annually. By converting this abundant, low-cost biomass into a value-added biocomposite material, this study investigates the feasibility of upcycling CS residues, thereby providing an industrial justification that strongly aligns with circular economy principles.

In parallel, polymer composite research has increasingly shifted toward numerical–experimental consistency as a prerequisite for engineering relevance [7]. Recent investigations of recycled PP/sawdust composites demonstrated that finite element (FE) models calibrated using true stress–true plastic strain data can reproduce tensile responses [18]. Broader reviews of wood- and lignocellulosic-based composites similarly emphasize that validated FE frameworks enable parametric design studies only when elastic constants, plastic behavior, and failure-relevant properties are aligned with experimental test configurations [1,2]. Related work on WPCs has further highlighted sensitivities to environmental conditions and failure modes, including temperature-dependent tensile degradation [19], mixed-mode bending fracture [20], and reliability under geometric and material variability [21]. Manufacturing-stage effects, such as drilling-induced damage and data-driven control of delamination, reinforce the need to integrate processing, mechanical testing, and FE validation into a coherent engineering workflow [22].

Accordingly, the present study investigates PP/CS biocomposites through a combined experimental and numerical framework. PP/CS samples were fabricated using two particle-size fractions (<100 µm and 100–250 µm), and length-based particle size distributions (D10, D50, D90) were quantified alongside complementary diameter statistics to rationalize particle-length differences associated with fine-sieve classification. Tensile and three-point bending tests were conducted to establish stiffness and strength trends as functions of particle size and filler loading. FE models of tensile and flexural behavior were then calibrated in ANSYS Workbench 2025 using experimentally derived true stress–true plastic strain data, with mesh design, boundary conditions, and convergence criteria reported explicitly in accordance with best practices for WPCs [1,18]. Where appropriate, results are benchmarked against related PP-based pruning-waste systems, thermoset wood-particle composites, untreated PLA biocomposites, and emerging natural fibers, without extrapolating across dissimilar matrices [6,13,16,17].

In the available literature, PP biocomposites reinforced with CS pruning waste particles have not yet been reported, and a mechanics-oriented assessment of such systems supported by FE validation remains absent. The present study integrates pruning-waste valorization with transparent constitutive calibration and FE-based numerical verification, thereby addressing both a materials gap and a methods gap. Specifically, it delivers experimentally informed and numerically validated tensile and flexural responses of PP-based biocomposites suitable for engineering-level analysis.

## 2. Materials and Methods

**Figure 1:**
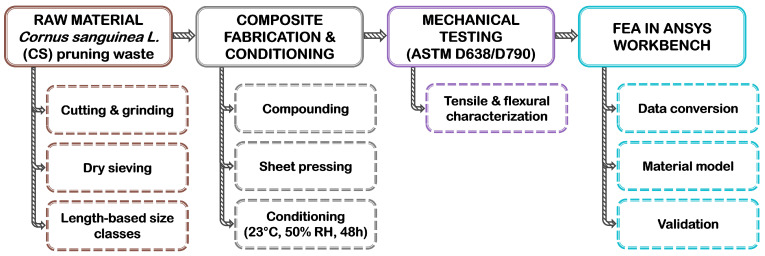
Schematic representation of the materials and methods workflow for PP/CS particle biocomposites.

Figure 1 illustrates the overall experimental and numerical workflow, which begins with CS pruning waste collection, grinding and sieving, length-based particle size characterization via morphology imaging, melt compounding, and sheet pressing. The subsequent phase involves specimen conditioning, ASTM D638 and D790 mechanical testing [23,24], data conversion for FE analysis, and final model validation in ANSYS Workbench.

### 2.1. Materials

Commercial PP pellets (LG Chem M1500, LG Chem Ltd., Seoul, Republic of Korea; melt flow index 16 g/10 min at 230 °C/2.16 kg; density 0.90 g cm^−3^) were used as the polymer matrix. The CS biomass was collected from the Aegean region of Türkiye, at an altitude of approximately 660 m above sea level, where the species is widely available and generates significant agricultural residue. The collected stem sections were separated, cleaned to remove surface impurities, and mechanically ground. The resulting material was sieved to obtain the desired particle size fractions. These lignocellulosic particles exhibited an irregular morphology with characteristic micrometer-scale dimensions, typical of mechanically processed natural fillers. Prior to composite fabrication, the CS particles were dried in a ventilated oven at 60 °C for 24 h to minimize moisture content, which was measured at 12.80% prior to processing, thereby ensuring stable conditions during melt compounding. The applied processing route was intentionally designed to be cost-effective and scalable without the need for chemical treatments. Consequently, the use of these untreated, size-selected lignocellulosic particles serves as a well-defined baseline reference condition for natural-filler-reinforced thermoplastic systems prior to any future interfacial modification [25].

### 2.2. Preparation, Size Classification, Morphology Imaging, and Statistics

Pruned branches were cut and ground using a laboratory grinder (Mertest LB160, Mertest Machine, Eskişehir, Türkiye) and subsequently dry-sieved manually through 100 µm and 250 µm meshes to obtain two length-based particle classes: under 100 µm (LF1) and between 100 and 250 µm (LF2). Owing to the ability of high-aspect-ratio fragments to pass through sieve apertures when aligned vertically, particle lengths occasionally exceeding the nominal cut-off are expected; such orientation-dependent passage is well documented for wood- and pruning-waste fillers [6,13]. To substantiate this interpretation, basic particle diameter statistics are reported for reference, while size classification is retained on a length basis.

Representative particles were imaged by Scanning Electron Microscopy (SEM), Carl Zeiss EVO 300VP, 3 kV, 100× (Carl Zeiss, Oberkochen, Germany). Prior to imaging, the samples were vacuum sputter-coated with a thin layer of gold, Quorum Q150 RES (Quorum, East Sussex, UK), to enhance surface conductivity and minimize electron beam–induced damage. The obtained micrographs are intended solely to illustrate particle morphology and size dispersion and should not be interpreted as composite fracture-surface analysis. Particle length and transverse Feret-type diameter were quantified using calibrated 2D image analysis. The resulting size distributions are summarized using the mean, standard deviation, coefficient of variation, and non-parametric empirical D10/D50/D90 percentiles. Outliers were purposely retained to preserve the true distribution tails arising from particle orientation and pass-through effects during mesh sieving.

The chemical composition of the CS particles was determined in accordance with standard TAPPI methods [26,27,28,29]. The analysis consisted of main steps including the removal of extractives, determination of lignin content, quantification of holocellulose, and measurement of α-cellulose. The results indicated that the CS filler consists of 39.10% α-cellulose, 24.14% hemicellulose, 26.25% lignin, 2.08% extractives, and 8.43% ash. Additionally, the moisture content of the particles prior to processing was determined to be 12.80%.

### 2.3. Biocomposite Fabrication and Sample Preparation

For each particle size, LF1 and LF2, the PP/CS formulations containing 0, 5, 10, 15, and 20 wt% of filler, detailed in Table 1, were initially compounded using a laboratory-type high-speed thermo-kinetic mixer (Gulnar Machine, Kayseri, Türkiye). Following the mixing, the blends were sheet-molded into solid composite plates using a heated–cooled hydraulic press (Gulnar Machine, Kayseri, Türkiye). To ensure uniform density and minimize internal voids, a critical consideration for dispersion phenomena often observed in pruning-waste/thermoplastic systems, a stepwise hot-pressing cycle was applied. Both the top and bottom heating plates were set to 180 °C, and the pressure was incrementally increased in three stages: 40 bar for 30 s, 70 bar for 30 s, and finally 120 bar for 30 s, resulting in a total hot-pressing time of 90 s. Immediately following this, a cold-pressing (cooling) phase was conducted under a constant pressure of 120 bar for 120 s. The total active press cycle time was 210 s.

Following hot-pressing, test specimens were extracted using high-precision cutting dies to prevent edge defects and ensure dimensional accuracy. Dog-bone specimens for tensile testing were die-cut according to ASTM D638 (115 mm overall length, 25 mm gauge length, 6 mm width, 3.2 mm thickness). Rectangular specimens for three-point bending followed ASTM D790 (50 × 15 × 3 mm). Prior to testing, all specimens were conditioned at 23 °C and 50% relative humidity for 48 h to ensure stable baseline behavior.

### 2.4. Mechanical Testing

All mechanical tests were conducted using a Shimadzu AGS-X universal testing machine (Shimadzu, Kyoto, Japan) equipped with a 5 kN load cell. Tensile tests were performed in accordance with ASTM D638 at a crosshead speed of 50 mm/min, while three-point bending tests followed ASTM D790 with a crosshead speed of 1 mm/min. For each material condition, a minimum of five replicate samples were tested to ensure statistical repeatability. The results are reported as the mean value ± standard deviation. The elastic modulus for all samples was derived directly from the load–displacement data obtained via the machine’s crosshead movement. While utilizing crosshead displacement provides a practical measure for observing relative stiffness changes among the different composite formulations, it is noted that this method may incorporate machine compliance and slight sample slippage. These factors should be taken into consideration when comparing absolute modulus values with other studies. Ultimately, the explicit reporting of the testing equipment, standard specifications, loading rates, and measurement methodology provides an ASTM-anchored basis for comparability with prior research on natural-filler polymer composites [1,6,13,18,19,30]. While the experimental procedures and sample dimensions strictly followed the above-mentioned ASTM standards, it should be noted for broader international reference that these methodologies are fundamentally comparable to their corresponding ISO standards, specifically ISO 527 [31] for tensile properties and ISO 178 [32] for flexural properties.

### 2.5. Data Reduction and Preparation for FEA

Engineering stress–strain data were exported from the TrapeziumX software Ver. 1.3.1 (Shimadzu, Kyoto, Japan). For tensile calibration, only the monotonic pre-necking portion of each curve was converted to true stress (Equation (1)) and true plastic strain (Equation (2)), consistent with isotropic hardening assumptions; post-necking data were excluded to avoid localization effects. The resulting curves were decimated to approximately 30 monotonic (ε^p^_true, σ_true) data pairs, preserving the yield and ultimate tensile strength regions to ensure numerical stability in tabular plasticity definitions within ANSYS Workbench (Static Structural). Flexural strength and initial and secant moduli were determined in accordance with ASTM D790.

(1) $$\sigma_{\mathrm{true}}=\sigma_{\mathrm{eng}}(1\;+\varepsilon_{\mathrm{eng}})$$

(2) $$\varepsilon_{\mathrm{true}}^{p}=\ln\;(1\;+\varepsilon_{\mathrm{eng}})\;-\frac{\sigma_{\mathrm{true}}}{E}$$

where σ is stress (MPa), ε is strain (dimensionless), subscript “eng” denotes engineering quantities, subscript “true” denotes true quantities, E is the elasticity modulus obtained from the initial linear region, and the superscript “p” indicates plastic strain.

### 2.6. Finite-Element Validation in ANSYS Workbench

Representative states, neat PP and selected filled compositions, were simulated in ANSYS Workbench 2025 to validate tensile and flexural responses. Each formulation was modeled as an equivalent homogeneous continuum (no explicit particle geometry), calibrated independently from its experimental response. Linear-elastic moduli (E_t_, E_f_) were taken directly from experimental results (Table 2), while Poisson’s ratio followed a literature-consistent PP baseline of ν = 0.35 with a ±0.05 sweep to assess solution sensitivity under both tension and bending [33,34]. This range covers the ν = 0.40 adopted for PP matrices in FE homogenization of PP/argan-shell aggregates [35] and is consistent with isotropic assumptions successfully applied in recycled PP/wood and PP/flax laminates modeled in ANSYS [36,37]. The isotropic value ν = 0.38 used in rPP/wood-particle simulations [18], together with reported ranges for comparable thermoplastic composites (ν ≈ 0.21–0.42) [38,39,40], further supports the selected range. In ANSYS, elastic–plastic response was implemented using an isotropic von Mises (J2) formulation with multilinear isotropic hardening (tabular plasticity), where the hardening curve is prescribed directly by the experimentally derived true stress–true plastic strain input already defined by Equations (1) and (2), rather than by an assumed closed-form hardening law. Elastoplastic tensile behavior was defined using experimentally derived true stress–plastic strain ($\varepsilon_{\mathrm{true}}^{p}$, $\sigma_{\mathrm{true}}$) data, with yield strength determined by the 2% offset criterion. Meshes and boundary conditions replicated the ASTM D638 and D790 sample geometries. For reproducibility, the sample was modeled as a 3D solid in Static Structural; geometric nonlinearity (large deflection) was enabled for three-point bending; supports and indenter were treated as rigid bodies; and stress–strain outputs were extracted at consistent locations (tension: mid-gauge; flexure: bottom mid-span tension surface) to match D638/D790 stress definitions for curve-level comparison.

Contact between the indenter and supports in three-point bending was modeled as frictional, with a sensitivity sweep coefficient μ = 0.0–0.3 (steps 0.1) to reflect frictionless to low-friction regimes commonly assumed for polymer–steel interfaces in validated WPCs and biodegradable sandwich-beam models [38,41]. Comparable orthotropic WPCs bending simulations reported realistic pre-crack responses using μ = 0.36–0.40 in LS-DYNA [20]; accordingly, the present μ range remains within the experimentally supported low-friction regime for polymer–metal contacts.

Similar friction coefficients (μ = 0.10–0.30) have been explicitly employed in bending FE analyses of sawdust/rPET and hybrid fiber composites [42,43], further supporting the representativeness of the selected sweep for dry polymer–metal interfaces. These modeling choices are consistent with established FE practices for natural-fiber and biocomposite systems under tensile and flexural loading, including orthotropy-aware formulations reported in related composite simulations [20,25,34,44,45]. The explicit orthotropic definitions adopted for PLA/jute and hemp-reinforced biocomposites [25,44] further support the mechanical-input framework applied here to the PP matrix and its fiber-filled variants. Because the present study targets particulate PP/CS composites at 0–20 wt% produced by grinding/sieving and compression molding, the macroscopic response was treated as quasi-isotropic; ν was therefore bracketed by sensitivity (0.30–0.40) rather than uniquely identified for each formulation. At higher filler contents (e.g., ≥30 wt% typical of decking-grade WPCs) and/or for more oriented reinforcements, ν and stiffness may become more formulation- and direction-dependent, and orthotropic or microstructure-informed strategies may be required. The FE model does not explicitly resolve particle distributions; dispersion/clustering and interfacial effects are captured implicitly through the formula-tion-specific experimental calibration, and directional cutting/testing was not performed.

## 3. Results and Discussion

### 3.1. Particle Size Distribution and Morphology of CS Fillers

**Figure 2:**
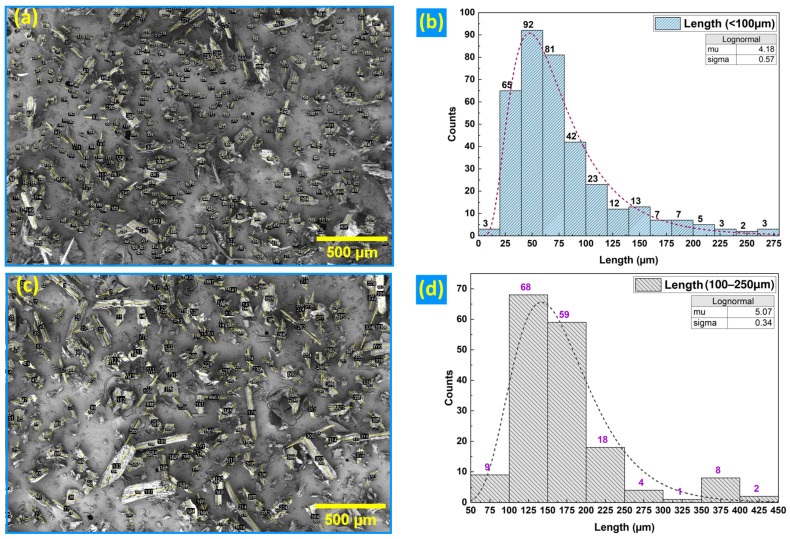
Morphology and length distributions of CS particles: (**a**) SEM, LF1, 100×; (**b**) LF1 length histogram with lognormal fit; (**c**) SEM, LF2, 100×; (**d**) LF2 length histogram with lognormal fit.

SEM micrographs reveal angular, splinter-like particles with rough surface textures (Figure 2a,c). The corresponding particle-length histograms (Figure 2d) were fitted with lognormal distributions to facilitate visualization of central tendency and dispersion.

#### 3.1.1. LF1 (<100 µm)

Length statistics (Figure 2b) show a mean of 77.1 µm (SD = 48.7 µm; CV = 0.63), a median of 63.9 µm, and empirical D10/D50/D90 values of 31.8/63.9/148.4 µm. The distribution is strongly right-skewed (skewness = 1.67) with pronounced tails (excess kurtosis = 2.82). The fitted lognormal model yielded parameters μ = 4.18 and σ = 0.57 in natural-log space, indicating a broad dispersion. A limited number of particle lengths exceeding 100 µm were observed; owing to the anisotropic particle geometry, passage through a <100 µm sieve is governed primarily by the smaller transverse dimension engaging the aperture, allowing slender particles to pass when favorably oriented. Diameter statistics for this fraction (mean = 36.5 µm; D90 = 59.9 µm) support this interpretation.

#### 3.1.2. LF2 (100–250 µm)

Length statistics (Figure 2d) indicate a mean of 170.1 µm (SD = 69.3 µm; CV = 0.41), with a median of 155.1 µm and empirical D10/D50/D90 values of 109.2/155.1/236.7 µm. The fitted lognormal distribution yielded parameters μ = 5.07 and σ = 0.34, reflecting a narrower length dispersion than that observed for LF1. Occasional particle lengths exceeding 250 µm were recorded and are attributed to orientation-dependent pass-through effects during sieving. Corresponding particle diameters were substantially smaller than lengths (mean = 61.2 µm; D90 = 95.9 µm), consistent with size classification governed by the limiting transverse cross-section of the sieve aperture. The combined use of empirical D10/D50/D90 metrics and SEM observations provides a controlled basis for examining particle-size effects on mechanical response in subsequent sections.

### 3.2. Mechanical Behavior in Tension and Bending

#### 3.2.1. Tensile Response of Neat PP and PP/CS Biocomposites

**Figure 3:**
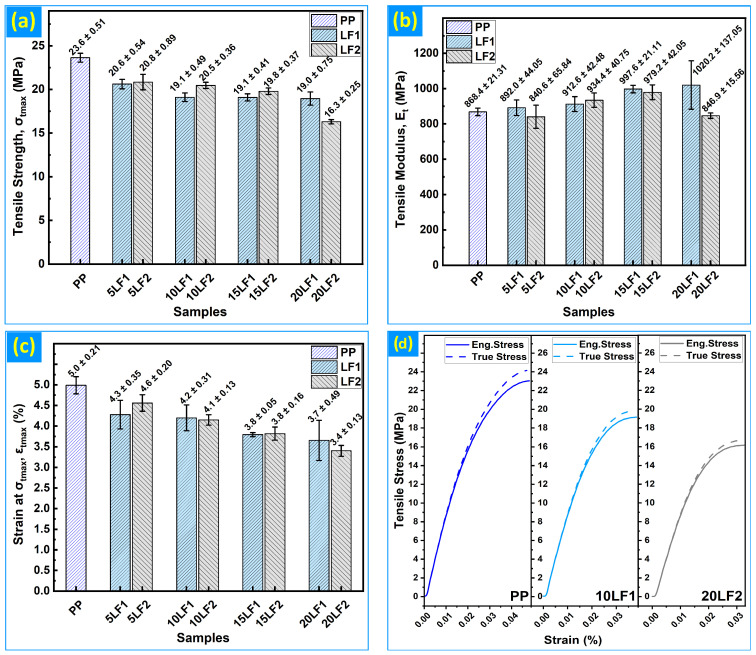
PP and PP/CS at 0–20 wt% with LF1 and LF2: (**a**) Tensile strength (σ_t,max_); (**b**) Tensile modulus (E_t_); (**c**) Tensile strain at maximum stress (ε_t,max_); (**d**) Representative tensile stress–strain curves: engineering (solid) and true (dashed).

Neat PP exhibited a tensile strength of 23.63 ± 0.51 MPa and a tensile modulus of 868 ± 21 MPa, with a maximum tensile strain (ε_t,max_) of 4.99 ± 0.21%. The incorporation of CS particles led to a systematic reduction in tensile strength at all filler loadings (Figure 3a). For the LF1, the maximum tensile stress (σ_t,max_) decreased by 12.8% at 5 wt%, 19.2% at 10 wt%, 19.2% at 15 wt%, and reached a total reduction of 19.8% at 20 wt% relative to neat PP. The LF2 exhibited a comparable strength reduction at 5–15 wt% (11.8–16.2%); however, at 20 wt% the LF2 biocomposite showed a pronounced decline to 16.30 ± 0.25 MPa (31.0% below neat PP), indicating a damaging interaction between particle size and high filler loading.

In contrast, stiffness generally increased with filler loading (Figure 3b). The tensile modulus E_t_ increased monotonically for the LF1 series, reaching a maximum of 1020 ± 137 MPa at 20 wt%, corresponding to a 17.5% increase relative to neat PP. The LF2 series exhibited more modest stiffness gains up to 15 wt% (12.8% increase at 15 wt%); however, at 20 wt% the modulus declined to 847 ± 16 MPa (2.5% below neat PP), consistent with a reduction in effective load transfer at high filler contents. Ductility decreased across all biocomposite formulations (Figure 3c), with the maximum tensile strain (ε_t,max_) dropped from 4.99% for neat PP to 3.65% at 20 wt% LF1 (26.8% reduction) and to 3.40% at 20 wt% LF2 (31.8% reduction).

This response, rising tensile modulus (E_t_) with concurrent reductions in tensile strength (σ_t,max_ and ε_t,max_), is characteristic of wood-like fillers in non-coupled polyolefins [34,45,46,47]. Similar behavior was reported for PP reinforced with pruned cherry-branch particles, where <100 µm fillers outperformed 100–250 µm fractions due to improved dispersion and fewer stress concentrators [6]. The present LF1–LF2 contrast, particularly the marked collapse in σ_t,max_ and E_t_ at 20 wt% LF2, aligns directly with this mechanism. Comparable strength losses and anisotropic strain responses at high loadings in starch-g-PP/kenaf biocomposites further confirm that particle orientation and matrix–filler adhesion govern stiffness–ductility trade-offs [48]. To visualize the full tensile response beyond single-point metrics, Figure 3d assembles representative engineering (solid lines) and true (dashed lines) σ–ε curves for neat PP, 10LF1 and 20LF2. The true curves are shown up to uniform elongation (pre-necking) and highlight differences in initial stiffness, yield behavior, and post-yield evolution, thereby motivating the FE analysis overlays discussed in the subsequent section.

Fracture-mechanics-guided analyses of extruded WPCs show that larger inclusions and imperfect interfacial bonding elevate local shear and stress triaxiality near the fracture-process zone, thereby promoting crack initiation and mixed-mode kinking [20]. Comparable stiffness–strength trade-offs are reported for PLA-based natural-fiber biocomposites where stiffness increases with fiber content but strength and ductility deteriorate beyond low loadings in the absence of interfacial treatment [17,25]. In contrast, interfacial modification is well known to restore strength while retaining stiffness, as demonstrated for PLA/wood and related biocomposites [49].

The present tensile results indicate that interfacial quality and particle morphology dominate failure behavior, and the LF1 network effectively contributes to small-strain stiffness. In contrast, high LF2 contents promote debonding and void-assisted fracture, consistent with morphologies reported for PP/wood particulate systems. FE–supported studies of fly ash/sisal and kenaf–coir biocomposites further show that stiffness gains from rigid fillers are accompanied by reduced deformation capacity as filler volume fraction increases [39,50]. Broader reports on biocomposites such as epoxy with wood particles [13] and polymer systems with dense inorganic particulates [30] also attribute strength losses at high filler levels to clustering and interfacial defects despite concurrent modulus gains.

#### 3.2.2. Flexural Response of Neat PP and PP/CS Biocomposites

**Figure 4:**
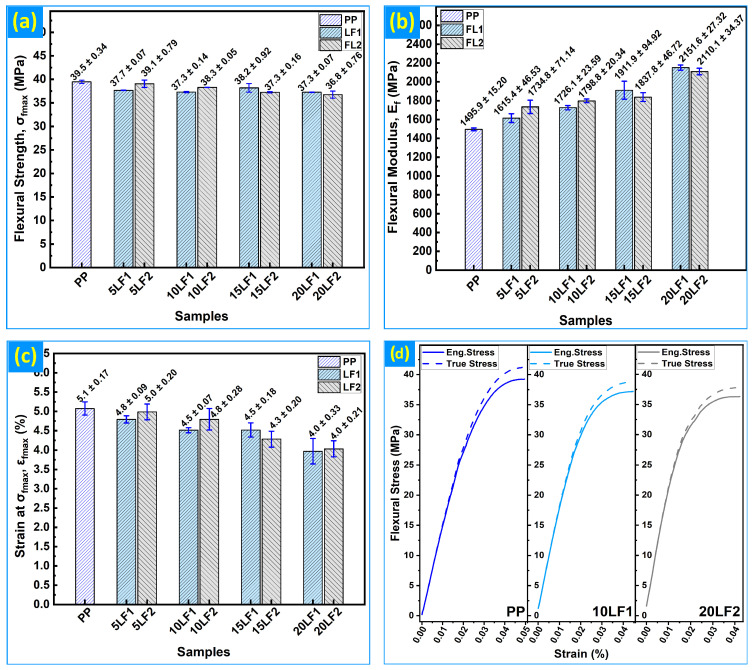
PP and PP/CS at 0–20 wt% with LF1 and LF2: (**a**) Flexural strength (σ_f,max_); (**b**) Flexural modulus (E_f_); (**c**) Flexural strain at maximum stress (ε_f,max_); (**d**) Representative flexural σ–ε curves: engineering stress (solid) and true stress (dashed).

In three-point bending, the maximum flexural stress (σ_f,max_) was markedly less sensitive to reinforcement than the tensile strength (σ_t,max_), Figure 4a. Relative to neat PP (39.5 ± 0.34 MPa), only modest reductions were observed for the biocomposites: −4.6% (5 wt% LF1), −5.5% (10 wt% LF1), −3.3% (15 wt% LF1) and −5.6% (20 wt% LF1), with LF2 tracking between −1.1% (5 wt%) and −6.9% (20 wt%). By contrast, the flexural modulus (E_f_) increased strongly and near-monotonically with filler loading (Figure 4b), reaching 2152 ± 27 MPa for 20 wt% LF1 (+43.8% vs. PP) and 2110 ± 34 MPa for 20 wt% LF2 (+41.1%). As illustrated in Figure 4c, flexural ductility (ε_f,max_) decreased progressively from 5.1 ± 0.17% (neat PP) to 3.97 ± 0.33% (20 wt% LF1, −21.8%) and 4.03 ± 0.21% (20 wt% LF2, −20.5%). Figure 4d shows representative flexural σ–ε curves for PP, 10LF1, and 20LF2, where engineering (solid) and true (dashed) responses are shown on the same axes, highlighting the steeper initial stiffness and earlier onset of nonlinearity in the biocomposites.

The pronounced stiffness amplification accompanied by only modest strength penalties in bending is fully consistent with the established behavior of WPCs [34,46,47,51]. The particle reinforcement effectively resists bending, which increases the flexural modu-lus (Ef). Furthermore, surface defects have little effect on the maximum flexural stress (σf,max) unless the particles begin to detach from the matrix [6]. Broad biocomposite literature supports this asymmetry: flexural stiffness gains often dominate even when tensile strength declines, because the compressive region of a bent beam is comparatively insensitive to microvoids and local decohesion [25]. FE analyses validated for PP/wood and marine biocomposites report similar trends, with increasing bending stiffness and failure governed primarily by matrix shear rather than fiber rupture [37,52]. Elastic–plastic FE analyses of high-density polyethylene-based WPCs likewise demonstrate monotonic increases in flexural modulus with increasing wood content [38]. More generally, highly filled polymer systems exhibit rising modulus and plateauing or declining strength at elevated filler levels due to agglomeration and porosity, as observed in polyester–WO_3_ and other particulate-filled polymer [30]. FE validation of rPET/wood systems and reliability analyses of WPCs decking further confirm that stiffness enhancement persists up to high filler loadings [21,42].

Across all formulations, the flexural modulus (E_f_) exceeds the tensile modulus (E_t_), as expected. Three-point bending imposes a through-thickness strain gradient that concentrates load in the outer fibers farthest from the neutral axis, which are less sensitive to internal defects; uniaxial tension, in contrast, averages strain over the full cross-section and is more affected by matrix–particle debonding and system compliance. In addition, ASTM D790 moduli are determined at very small deflections with one surface in compression, suppressing microcrack opening, whereas ASTM D638 tensile moduli integrate axial strain over a longer gauge length. Accordingly, E_f_ > E_t_ is observed for all compositions (e.g., PP ≈ 1.50 vs. 870 MPa; 20 wt% LF1 ≈ 2.15 vs. 1020 MPa).

#### 3.2.3. Effect of Particle Loading and Particle Size

Across the 0–20 wt% CS range, stiffness increases while strength and ductility decrease, with the penalty markedly more severe in tension than in bending, consistent with untreated lignocellulosic particulates in thermoplastics [6,20,25,45,46,51]. Particle-size effects are limited up to 15 wt% but become decisive at 20 wt%, where the finer fraction (LF1) outperforms LF2. This trend is consistent with reports that larger particles intensify stress concentrations and promote early interfacial debonding [6,20], and that bark- or mineral-rich fractions underperform wood particles at higher loadings due to poorer wetting and increased microvoid content [13]. Comparable observations in epoxy/sisal–fly ash composites further show that rigid microfillers increase modulus at the expense of strain to failure as voids accumulate [39].

It is important to note that the composites in this study were fabricated using compression molding. This processing technology typically results in a random, planar distribution of fibers, leading to largely isotropic mechanical properties without significant fiber orientation. Unlike injection molding, which aligns fibers and enhances directional reinforcement efficiency, the chosen method inherently limits the maximum achievable mechanical performance. Consequently, this isotropic nature explains why variations in filler length or particle shape exhibited only a marginal effect on the overall mechanical behavior of the WPCs in this study.

From a design perspective, stiffness-critical components, like interior panels and decking facings, can benefit from validation 15–20 wt% LF1, which provides substantial gains in flexural modulus while maintaining σ_f,max_ close to that of neat PP [21,42]. For applications requiring a more balanced tensile response, ≤10–15 wt% LF1 limits losses in σ_t,max_, with additional recovery achievable through compatibilization or surface treatments and low-void consolidation routes (e.g., injection molding or vacuum-assisted processing) [5,17,49,53]. Designs subjected to temperature fluctuations should also account for the pronounced thermal softening of WPCs, which reduces both strength and modulus at a given composition [19].

### 3.3. Finite-Element Setup and Validation

#### 3.3.1. Geometry and Meshing

**Figure 5:**
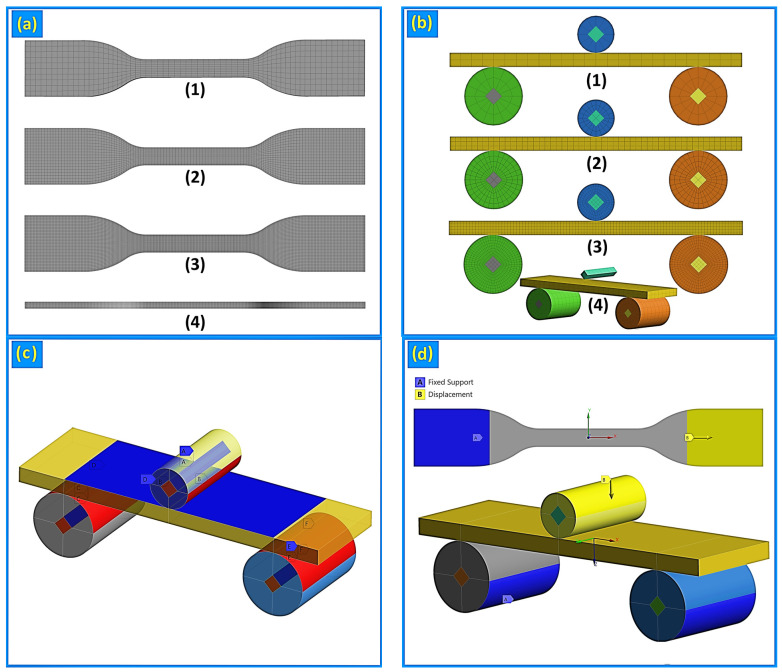
FE model setup: (**a**) tensile sample meshing and (**b**) three-point bending meshing at corresponding refinements, (1) coarse, (2) medium, (3) fine, with (4) a detailed contact view; (**c**) contact definitions for the flexural configuration; (**d**) applied boundary conditions for tensile and flexural simulations.

The tensile and flexural samples were discretized using a structured MultiZone meshing strategy with sweepable hexahedral elements (solid elements, program-controlled order), ensuring a uniform element distribution within the tensile gauge section and the mid-span region under bending. Representative meshes for the tensile and flexural configurations are presented in Figure 5a,b. Three mesh densities, coarse (2.0 mm), medium (1.0 mm), and fine (0.5 mm), were evaluated to assess mesh sensitivity for one representative tensile case (neat PP, mid-gauge stress) and one flexural case (neat PP, bottom mid-span stress on the tension side). Mesh quality was verified using maximum skewness, Gauss-point Jacobian ratio, and maximum aspect ratio. The corresponding convergence metrics and mesh-quality extrema are summarized in Table 3. For the flexural models, average skewness decreased systematically with mesh refinement (from approx. 0.166 to 0.081), while the maximum skewness remained constant at 0.50 across all mesh levels, indicating stable element shape quality throughout refinement.

For tension, the difference between medium and fine meshes was negligible (<0.01%), indicating mesh independence already at the medium level (Table 3). In flexural simulations, the coarse mesh underestimated the peak bending stress, whereas the medium mesh reduced this deviation to below 4% while requiring approximately threefold less computational cost than the fine mesh. Considering the mesh-quality extrema and the minimal stress deviation relative to the fine-mesh reference, the medium mesh (1.0 mm) was selected for all subsequent tensile and flexural analyses as an optimal compromise between accuracy and computational efficiency.

#### 3.3.2. Boundary Conditions and Contacts

Boundary conditions and contact definitions followed ASTM D638 (tension) and ASTM D790 (three-point bending) prescriptions and are illustrated in Figure 5c,d. In tension, one grip end was fully constrained while axial displacement was prescribed at the opposite end to ensure stable control of the nonlinear response. In flexure, the sample rested on two rigid supports and was loaded via a rigid indenter through frictional surface-to-surface contact. Stress evaluation locations were defined at the tensile mid-gauge and at the bottom mid-span surface in bending (tension side), ensuring physically meaningful stress extraction.

#### 3.3.3. Stress–Strain Overlays

**Figure 6:**
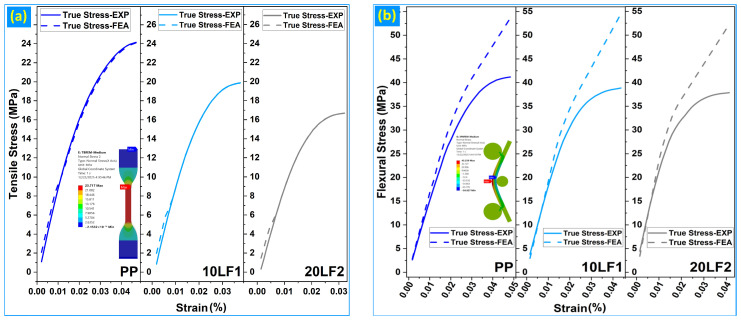
Experimental and FE true stress–strain overlays for PP, 10LF1, and 20LF2: (**a**) in tension; (**b**) in three-point bending.

Figure 6a,b compare experimental and FE stress–strain responses for three representative compositions: neat PP, 10LF1, and 20LF2. These cases span the unfilled matrix, an intermediate filler content exhibiting stable behavior, and the most critical formulation characterized by pronounced nonlinearity, thereby capturing the full mechanical contrast of the system. For both tensile and flexural loading, stress–strain overlays are presented using true stress–true strain representations for visualization, whereas all quantitative accuracy metrics were evaluated in engineering stress–strain space following consistent back-conversion of FE outputs [18]. Experimental and numerical curves were interpolated and sampled at identical strain coordinates, ensuring one-to-one data correspondence and eliminating sampling-induced bias [54].

The tensile stress–strain overlays (Figure 6a) show excellent agreement between experiment and FE predictions across the full strain range for all three compositions. The numerical responses closely reproduce both the initial elastic slope and the subsequent nonlinear hardening behavior, with only minor deviations observed near the terminal region. This strong curve-level agreement is corroborated quantitatively by the low mean absolute percentage error (MAPE), high coefficients of determination (R^2^), small stress discrepancies at ε = 1% and 2%, and strength ratios (σ_max,FE_/σ_max,Exp_) approaching unity, as summarized in Table 4.

The flexural stress–strain overlays (Figure 6b) exhibit a distinct response. While agreement between experiment and FE predictions is strong in the early and intermediate deformation regimes, systematic divergence appears at higher strains, particularly beyond approximately 50% of the experimental maximum flexural stress (σ_max,Exp_). This deviation, most evident in neat PP and emphasized in 20LF2, is attributed to contact nonlinearity, evolving stress localization, and sensitivity to frictional assumptions at large deflections, as widely reported for polymer and WPCs bending simulations [41,42,51,55]. Consequently, numerical accuracy metrics for flexure were evaluated over the 0–50% σ_max,exp_ domain, where the mechanical response remains dominated by bending stiffness rather than contact-dominated nonlinearities [38].

The resulting MAPE_0–50_%, R^2^_0–50_%, pointwise stress errors at ε = 0.5% and 1%, and strength ratios are summarized in Table 5. Overall, the stress–strain overlays confirm that the FE framework accurately reproduces the tensile response over the full deformation range and the flexural response within the mechanically meaningful pre-contact-dominated regime. The observed divergence at large flexural strains further supports the use of a truncated evaluation window and is consistent with established validation practices in polymer and biocomposite FE studies [18,51,52,53].

#### 3.3.4. Parity Analysis

**Figure 7:**
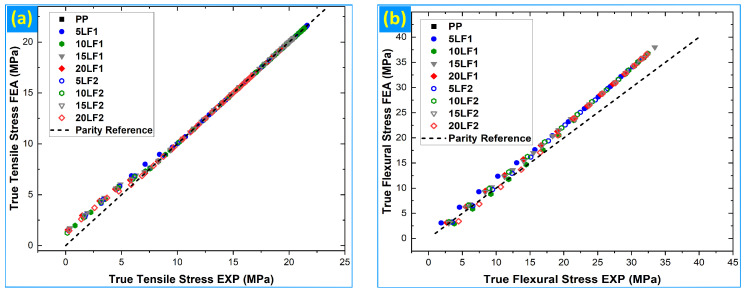
Parity plots comparing experimental and FE stresses for all compositions: (**a**) tensile stress over the full experimental stress range; (**b**) flexural stress within the 0–50% σ_max,exp_ domain.

Figure 7a and Figure 7b present parity plots comparing finite-element and experimental stresses for all nine compositions in tension and flexure, respectively, with the experimental stress on the abscissa and the simulated stress on the ordinate. In contrast to the curve-level overlays discussed previously, the parity analysis assesses global agreement relative to the ideal 1:1 correspondence across the full stress domain for tensile loading and the truncated 0–50% σ_max,exp_ domain for flexural loading [51].

For the tensile response (Figure 7a), the data cluster tightly around the 1:1 parity line for all formulations, indicating excellent global agreement between FE predictions and experimental measurements. This is quantified by low root-mean-square errors (RMSE ≈ 0.40–0.50 MPa), near-zero bias (|Bias| ≤ 0.21 MPa), and very high Lin’s concordance correlation coefficients (CCC ≈ 0.996–0.998) across the entire dataset (Table 6). Physically, the low RMSE reflects minimal dispersion of numerical predictions about the parity line, while the near-zero bias confirms the absence of systematic over- or under-prediction by the model. The CCC values approaching unity demonstrate strong concordance, capturing both the tight dispersion and the near-unity slope of the stress correspondence. These parity-based CCC values should be distinguished from the curve-level R^2^ reported earlier: while R^2^ evaluates how well the simulated curve follows the shape of the experimental stress–strain trajectory, CCC here directly measures agreement with the 1:1 parity condition, incorporating both correlation and absolute accuracy.

The flexural parity plot (Figure 7b) shows a broader, yet still coherent, distribution of points around the 1:1 line within the 0–50% σ_max,exp_ domain. Correspondingly, RMSE values are higher (≈2.4–2.8 MPa) and positive bias values (≈1.6–2.6 MPa) are consistently observed, indicating a modest systematic over-prediction of flexural stress by the simulations in this regime. Nevertheless, CCC values remain high (≈0.96–0.97) for all compositions (Table 7), confirming strong overall agreement once contact- and large-deflection-dominated regions are excluded. The increased RMSE relative to tension reflects the greater sensitivity of bending response to contact conditions and local stress redistribution, whereas the preserved CCC demonstrates that the numerical model reproduces the experimental ranking and proportionality of stresses with high fidelity in the mechanically meaningful range [37,43,51].

When considered together, the parity analysis corroborates the curve-level validation by demonstrating that the FE framework delivers both low scatter and negligible systematic bias in tension over the full stress range, and reliable global agreement in flexure within the pre-contact-dominated regime. The combined use of RMSE, bias, and CCC thus provides a complementary and physically transparent assessment of numerical–experimental agreement beyond traditional curve-fit metrics [1,56].

### 3.4. Sensitivity Analysis

**Figure 8:**
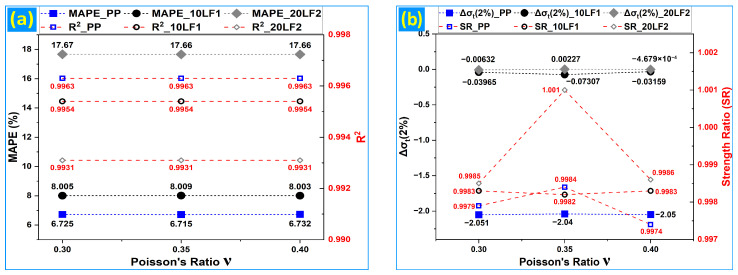
Tensile sensitivity to Poisson’s ratio (ν): (**a**) MAPE and R^2^ vs. ν; (**b**) Δσ_t_(2%) and strength ratio vs. ν.

Figure 8a,b summarize the sensitivity of the validated finite-element framework to Poisson’s ratio (ν) in tension and to the contact friction coefficient (μ) in three-point bending, using representative compositions (PP, 10LF1, and 20LF2).

Under tensile loading, varying ν across 0.30–0.40 produces essentially invariant global and local agreement metrics for each material state: MAPE and R^2^ remain unchanged to within numerical noise, and the stress error at ε = 2% (Δσ_t_(2%)) stays close to zero for the composite formulations, with strength ratios tightly clustered around unity [33,34,35,40]. Mechanically, this insensitivity is expected because, under uniaxial tension of an isotropic solid, the axial stress–strain response is governed primarily by the imposed elastoplastic constitutive relation, while ν mainly controls lateral contraction and the associated transverse strain field. Within the tested interval, its influence on the axial stress trajectory is therefore secondary [18,33]. The slightly higher tensile MAPE observed for the most nonlinear formulation (20LF2) reflects the greater curvature and localization tendency of that response rather than a ν-driven instability, consistent with the near-constant R^2^ and peak-strength ratios across ν.

**Figure 9:**
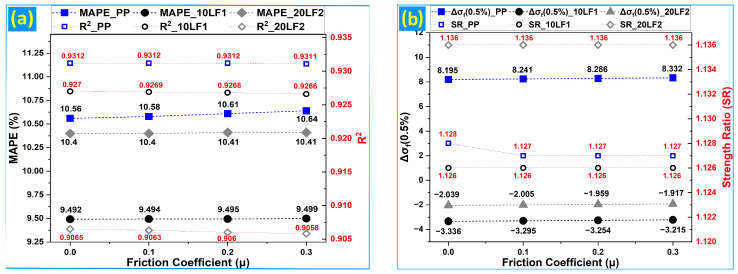
Flexural sensitivity to friction coefficient (μ): (**a**) MAPE_0–50%_ and R^2^_0–50%_ vs. μ; (**b**) Δσ_f_ (0.5%) and strength ratio vs. μ.

Under flexural loading (Figure 9a,b), sweeping μ from 0.0 to 0.3 over the pre-contact-dominated 0–50% regime leads to similarly modest changes in global metrics. Both MAPE and R^2^ change minimally for each representative composition, indicating that the overall stress–strain response is robust to low-friction assumptions within this range [41,43,51,55]. The local strain-level metric Δσ_f_ (0.5%) exhibits a systematic but small drift with increasing μ, consistent with friction influencing early load transfer and the partitioning of reaction forces between indenter and supports. However, the persistence of nearly constant strength ratios across μ indicates that peak-response scaling within the truncated domain is not controlled by contact friction for the present setup [37,38,42,51]. The sign and magnitude of Δσ_f_ (0.5%) further indicate that μ perturbs the early bending response in a material-dependent manner, consistent with differences in stiffness and nonlinearity among PP, 10LF1, and 20LF2. Overall, the sensitivity results demonstrate that ν and μ can be treated as secondary parameters within the selected ranges for curve-level validation. MAPE reflects the stability of global agreement, R^2^ confirms preservation of the stress–strain shape, Δσ quantifies localized sensitivity at low strain where design-relevant stiffness is inferred, and the strength ratio verifies consistency of peak-response predictions across the parameter sweeps [1,41].

## 4. Conclusions

This work provides a mechanics-focused, experimentally grounded, and FE–validated assessment of PP biocomposites reinforced with CS particles, establishing explicit links between particle size, filler content, deformation mode, and predictive capability. Within the present literature, it represents the first systematic evaluation of PP/CS systems using coupled mechanical testing and constitutive calibration, enabling interpretation of measured responses within an engineering modeling framework.

Experimentally, CS particle incorporation increased stiffness while reducing tensile strength and ductility, with a pronounced interaction between particle size and filler loading. The finer particle fraction produced a monotonic increase in tensile modulus up to 20 wt%, whereas the coarser fraction led to mechanically unfavorable behavior at high loading, culminating in a marked strength reduction at 20 wt%. In three-point bending, reinforcement exerted a weaker influence on flexural strength than in tension, while producing substantial increases in flexural modulus for both size classes. These trends indicate that PP/CS formulations are most suitable for stiffness-governed, semi-structural applications where modulus enhancement is prioritized and moderate strength retention is acceptable.

From a modeling perspective, FE models calibrated using experimentally derived true stress–true plastic strain data reproduced tensile responses with strong curve-level agreement across the full strain range and consistent parity-line correspondence across all compositions. This level of agreement supports the use of such calibrated constitutive models for predictive tensile analysis of PP-based biocomposites. In flexure, numerical predictions remained reliable within the mechanically relevant, pre–contact-dominated regime, while deviations at larger deflections underscore the increasing influence of contact nonlinearity, stress redistribution, and frictional assumptions inherent to three-point bending simulations. Sensitivity analyses further showed that Poisson’s ratio within the range ν = 0.30–0.40 plays a secondary role in uniaxial tensile response under isotropic assumptions, and that variations in friction coefficient (μ = 0.0–0.3) exert only a limited effect on global flexural agreement within the truncated regime, with observable influence confined to early-stage load transfer.

Overall, the results demonstrate that pruning-waste-derived CS particles can be used to tailor the stiffness of PP composites, while clearly defining the performance limits imposed by untreated particle–matrix interfaces, particularly for coarser particles at elevated filler contents. Future studies should therefore focus on interfacial modification and dispersion control to mitigate strength loss without sacrificing modulus, and on extending numerical validation into regimes dominated by contact, damage initiation, and progressive failure. Such developments are required to support reliable, model-informed design of PP/CS biocomposites under broader loading and environmental conditions.

## Figures and Tables

**Table 1:** Formulation matrix (weight fractions, wt%).

Code	Particle Size	PP	CS
PP	—	100	0
5LF1	<100 µm	95	5
10LF1	<100 µm	90	10
15LF1	<100 µm	85	15
20LF1	<100 µm	80	20
5LF2	100–250 µm	95	5
10LF2	100–250 µm	90	10
15LF2	100–250 µm	85	15
20LF2	100–250 µm	80	20

**Table 2:** Material properties used in FEA models (Et, Ef, ν sweep, μ sweep, and plastic true-curve reference including tensile yield strength (σyt), flexural yield strength (σyf) in MPa).

Code	Et (MPa)	Ef (MPa)	Plastic True Curve Reference
PP	868.4 ± 21.3	1495.9 ± 15.2	At σyt = 16.5/σyf = 30.19
5LF1	892.0 ± 44.0	1615.4 ± 46.5	At σyt = 16.3/σyf = 27.35
5LF2	840.6 ± 65.8	1734.8 ± 71.1	At σyt = 16.3/σyf = 28.50
10LF1	912.6 ± 42.5	1726.1 ± 23.6	At σyt = 15.6/σyf = 30.50
10LF2	934.4 ± 40.8	1798.8 ± 20.3	At σyt = 16.0/σyf = 28.50
15LF1	997.6 ± 21.1	1911.9 ± 94.9	At σyt = 15.6/σyf = 29.70
15LF2	979.2 ± 42.0	1837.8 ± 46.7	At σyt = 15.6/σyf = 29.30
20LF1	1020.2 ± 137.0	2151.6 ± 27.3	At σyt = 15.1/σyf = 29.00
20LF2	846.9 ± 15.6	2110.1 ± 34.4	At σyt = 14.3/σyf = 29.20

**Table 3:** Mesh convergence summary for representative tensile and flexural cases (neat PP).

Metric	Tensile (Mid-Gauge Stress)		
	Coarse	Medium	Fine
Element size (mm)	2.0	1.0	0.5
No. of elements	1300	7011	45,600
σmax (MPa)	24.071	24.069	24.068
Δ vs. fine (%)	+0.01	+0.00	0.00
Max skewness	0.36266	0.37247	0.37808
Max Jacobian ratio (Gauss)	1.00	1.00	1.00
Max aspect ratio	3.2353	3.1667	3.1667
	Flexural (bottom mid-span stress)		
Element size (mm)	2.00	1.00	0.50
No. of elements	1048	6960	51,960
σmax (MPa)	38.696	41.170	42.849
Δ vs. fine (%)	+9.69	+3.92	0.00
Max skewness	0.50	0.50	0.50
Max Jacobian ratio (Gauss)	1.00	1.00	1.00
Max aspect ratio	3.75	4.00	4.00

**Table 4:** Tensile numerical accuracy metrics.

Code	MAPE (%)	R^2^	Δσ (1%) (%)	Δσ (2%) (%)	Strength Ratio
PP	6.715	0.9963	4.73	−2.04	0.9984
5LF1	17.538	0.9951	3.80	−0.06	0.9979
10LF1	8.009	0.9954	−0.13	−0.07	0.9982
15LF1	15.872	0.9940	−0.02	−0.01	0.9989
20LF1	29.420	0.9929	−0.08	−0.10	0.9984
5LF2	18.895	0.9958	0.02	−0.03	1.0004
10LF2	28.493	0.9962	−0.07	−0.07	1.0000
15LF2	17.811	0.9946	−0.01	−0.11	0.9980
20LF2	17.664	0.9931	−0.02	0.00	1.0007

**Table 5:** Flexural numerical accuracy metrics.

Code	MAPE_0–50% (%)	R^2^_0–50%	Δσ (0.5%) (%)	Δσ (1%) (%)	Strength Ratio
PP	9.504	0.9472	8.20	10.80	1.3018
5LF1	26.002	0.8873	24.33	12.20	1.4025
10LF1	7.141	0.9819	−3.34	5.34	1.4021
15LF1	8.930	0.9460	9.19	11.32	1.4417
20LF1	12.792	0.9302	11.74	11.57	1.3725
5LF2	6.348	0.9541	2.57	9.54	1.3966
10LF2	10.891	0.9318	10.50	11.52	1.4497
15LF2	9.048	0.9445	9.92	11.04	1.4616
20LF2	7.919	0.9782	−2.04	10.50	1.3804

**Table 6:** Tensile parity metrics.

Sample	RMSE (MPa)	Bias (MPa)	CCC
PP	0.425	−0.017	0.998
5LF1	0.462	0.207	0.997
10LF1	0.397	0.152	0.998
15LF1	0.465	0.178	0.997
20LF1	0.499	0.187	0.996
5LF2	0.429	0.163	0.998
10LF2	0.404	0.151	0.998
15LF2	0.450	0.171	0.997
20LF2	0.422	0.164	0.996

**Table 7:** Flexural parity metrics.

Sample	RMSE (MPa)	Bias (MPa)	CCC
PP	2.378	2.050	0.971
5LF1	2.732	2.583	0.963
10LF1	2.384	1.626	0.971
15LF1	2.817	2.459	0.964
20LF1	2.630	2.356	0.965
5LF2	2.655	2.152	0.965
10LF2	2.733	2.414	0.963
15LF2	2.539	2.195	0.968
20LF2	2.712	1.959	0.963

## Data Availability

The original contributions presented in this study are included in the article. Further inquiries can be directed to the corresponding author.
